# Eye Movement Dynamics Differ between Encoding and Recognition of Faces

**DOI:** 10.3390/vision3010009

**Published:** 2019-02-12

**Authors:** Joseph M. Arizpe, Danielle L. Noles, Jack W. Tsao, Annie W.-Y. Chan

**Affiliations:** 1 Department of Neurology, University of Tennessee Health Science Center, Memphis, TN 38163, USA; 2 Children’s Foundation Research Institute, Le Bonheur Children’s Hospital, Memphis, TN 38103, USA; 3 Science Applications International Corporation (SAIC), Fort Sam Houston, TX 78234, USA; 4 School of Medicine, University of Tennessee Health Science Center, Memphis, TN 38163, USA; 5 Department of Anatomy & Neurobiology, University of Tennessee Health Science Center, Memphis, TN 38163, USA; 6 Memphis Veterans Affairs Medical Center, Memphis, TN 38104, USA; 7 Department of Radiology, University of Tennessee Health Science Center, Memphis, TN 38163, USA; 8 Department of Life Sciences, Centre for Cognitive Neuroscience, Division of Psychology, Brunel University London, London, UB8 3PH, UK

**Keywords:** face, eye-movement, encoding, recognition, gaze, fixation, identification

## Abstract

Facial recognition is widely thought to involve a holistic perceptual process, and optimal recognition performance can be rapidly achieved within two fixations. However, is facial identity encoding likewise holistic and rapid, and how do gaze dynamics during encoding relate to recognition? While having eye movements tracked, participants completed an encoding (“study”) phase and subsequent recognition (“test”) phase, each divided into blocks of one- or five-second stimulus presentation time conditions to distinguish the influences of experimental phase (encoding/recognition) and stimulus presentation time (short/long). Within the first two fixations, several differences between encoding and recognition were evident in the temporal and spatial dynamics of the eye-movements. Most importantly, in behavior, the long study phase presentation time alone caused improved recognition performance (i.e., longer time at recognition did not improve performance), revealing that encoding is not as rapid as recognition, since longer sequences of eye-movements are functionally required to achieve optimal encoding than to achieve optimal recognition. Together, these results are inconsistent with a scan path replay hypothesis. Rather, feature information seems to have been gradually integrated over many fixations during encoding, enabling recognition that could subsequently occur rapidly and holistically within a small number of fixations.

## 1. Introduction

Eye movement studies have helped in the investigation of the different visual information sampling mechanisms involved in various cognitive processes concerning facial perception, such as identity recognition [1,2], matching [3,4], emotional expression identification [5,6], and other-race identification [7,8,9,10,11], among others. Although some prior studies have examined the eye movement dynamics during facial identity recognition and the functional significance of these dynamics, the exact relationship between eye movements during facial identity encoding and those in recognition remain to be elucidated. The present study aims to help fill this gap by investigating the difference and relationship between the visual processing mechanisms of facial encoding and recognition.

Prior eye movement evidence indicates that two fixations suffice for optimal facial recognition and that initial fixations correspond to an optimal location for facial identification information sampling. In one relevant study [2], participants were asked to study a series of faces for three seconds each. Participants were then required to perform an old/new facial recognition task on a series of faces, half of which were those previously studied. During the test phase, the number of permissible fixations across trials was varied (1, 2, 3, or unrestricted fixations). Discrimination performance in the test phase was greater for two permissible fixations than for one, but did not increase beyond two fixations, thus revealing that face recognition is optimal after only two fixations. An additional control condition confirmed that the advantage for two fixations over one was not merely due to increased viewing time, thus indicating how functionally important the second fixation is for face recognition. Another study investigated the functional significance of the location of initial fixations [1]. Participants in that study were required to identify each of a series of 125 rapid presentations (350 or 1500 ms) of faces as one of ten possible identities. The preferred location of the initial fixation tended to land over a featureless location just below the eyes, which, according to a Bayesian ideal observer model, corresponds to a location that is optimal for facial information integration. Indeed, when participants were forced to fixate at other locations while performing the task, group average identification performance decreased. Thus, that preferred initial fixation location was also the functionally optimal location for face identification. An additional study [12] further revealed that the preferred and optimal location was consistent between groups of observers of different races (though for results consistent with differences between races, also see [11,13]). A different study [14] also reported findings consistent with the functional significance of initial fixations. Specifically, it reported that initial fixations to upright faces tended to fall on or near the eyes, that recognition performance was lower when freely made initial fixations landed on the mouth compared to when they landed on the eyes, and that recognition performance was lower when the mouth was cued before stimulus presentation compared to when the eyes were cued. Taken together, these studies reveal that sampling of many facial features via dispersed fixation is not necessary for face recognition, but rather that faces are recognized rapidly and putatively in a holistic manner.

What remains unclear is how facial identity representations are formed during encoding and how these representations relate to the few functionally relevant eye movement dynamics measured during recognition. A study of simple pattern recognition [15] reports that participants usually followed the same scan path between encoding and recognition for a given visual pattern. This was taken to suggest that recognition could function through the replaying of eye movements performed during encoding. If this is so, visual memory traces formed during encoding could each be judged against the visual percept at recognition through perhaps even fairly retinotopically specific perceptual comparisons. This scan path replay hypothesis was first proposed several decades ago. The correlation between encoding and recognition scan path sequences has since been conceptually replicated in other studies that have used various visual stimuli and that have further indicated that low-level image properties and modeled saliency mapping seem to have more limited influence than do top-down factors on the scan paths observed [16,17,18,19,20,21,22,23,24,25,26]. A correlation between encoding and recognition scan paths, even if well replicated, does not necessarily imply any causal or functional relevance to recognition, however. The only investigation into the functional relevance of replayed eye movement sequences for recognition has been interpreted as challenging the notion of the functional necessity of scan path replay for recognition. In that study of scene recognition [27], participants studied visual scenes with freely made eye-movements; however, during the recognition phase of the experiment, the participants were shown only patches of scenes. The centers of these scene patches corresponded either to the locations of their own prior encoding fixations or to those of other participants’. Importantly, forcing each participant to view scene patches centered on another participant’s encoding phase fixations did not reduce recognition performance compared to viewing scene patches reflecting one’s own eye movements. The possibility that the spatial patterns of gaze for the stimuli could have been similar among participants in that experiment, however, casts doubt on the result as definitive evidence against the functional necessity of scan path replay for visual recognition.

**Figure 1:**
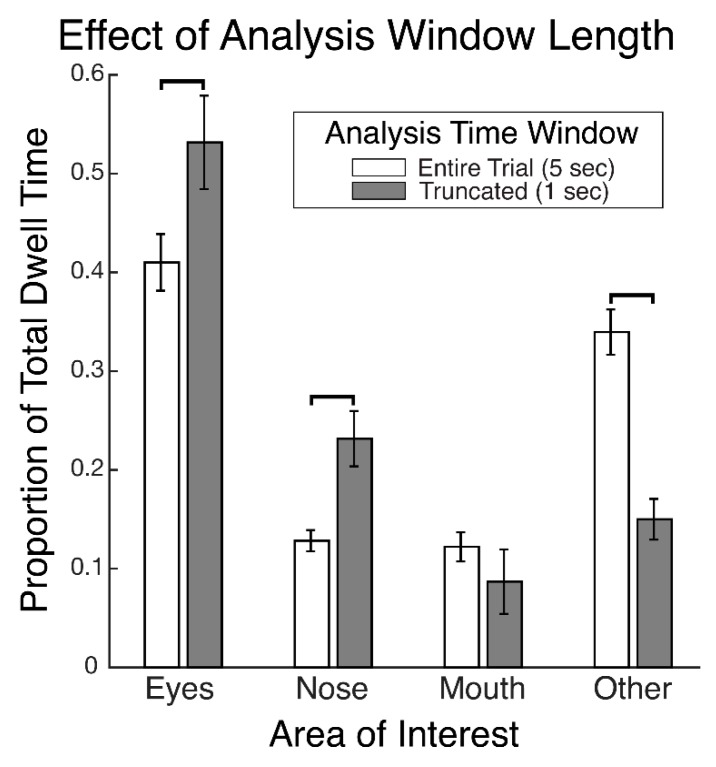
Effect of analysis time window length on the proportions of total dwell time across facial features. The comparison between five seconds of facial encoding (white bars) and the same, non-independent, data truncated to just the first second of encoding (gray bars) revealed highly significant differences in proportion of dwell time on the eyes (*p* = 0.0003), nose (*p* = 0.000027), and “other” facial areas of interest (*p* = 9 × 10^−14^). The difference for the mouth area was only a trend (*p* = 0.10). The pattern and magnitude of these differences match those interpreted as differences between encoding and recognition in Henderson, Williams, & Falk, 2005 [28]. Our illustration, instead, suggests that the differences reported by Henderson and colleagues are likely largely, if not entirely, analytic artefacts of the inconsistent time window lengths applied between their encoding and recognition phases.

A study of face recognition that is relevant to this question of the functional necessity of scan path replay [28] reports that the proportions of time spent gazing at different facial regions during recognition did not differ between faces that had been encoded with fixation restricted to a central facial location and those that had been encoded with freely made fixations. This suggests that participants were not replaying gaze patterns at recognition which reflected any restriction of gaze during encoding. That study further reports that gaze patterns during face recognition were more restricted to the eye and nose regions compared to the patterns made during the free viewing encoding condition, suggesting that gaze patterns differed between encoding and recognition of faces. The gaze time proportions were calculated over the entire stimulus viewing periods. These were approximately 2 s long, on average, during recognition and were 10 s long during encoding. Therefore, given that only the first two fixations would putatively have been the most functionally relevant for recognition, this means that many functionally superfluous fixations were included in the analysis, thereby possibly obscuring a modulation of the functionally relevant gaze patterns at recognition that could have reflected the restricted gaze at encoding. Further, the time windows over which gaze was analyzed between encoding and recognition were not equivalent, and so the relative pattern of differences may have been due to the time window length rather than due to the experimental phase. In a preview of the data from our present study, Figure 1 demonstrates a clear empirical confirmation and exemplification of precisely such an analysis-dependent artefact that can be attributed entirely to the difference in analysis time window length (see also “Areas of Interest Analysis” in Results). For these reasons, it is still unclear whether scan path replay occurs between face encoding and recognition and is functionally relevant to recognition. Further, if replay does not occur, it is unknown what gaze dynamic is instead at play between encoding and recognition of faces.

Those limitations with respect to the question of eye-movement recapitulation aside, the main aim of the study just described in the previous paragraph was to determine whether eye-movements functionally facilitate the encoding of faces. That study provided strong and valuable evidence in support of this. Specifically, those faces that had been encoded with fixation restricted to a central facial location were later recognized less accurately (52.5%) than those that had been encoded with freely made fixations (81.3%). This would seem to imply that optimal face encoding functionally requires a dispersed sampling of the specific visual features of a face through multiple fixations, in contradistinction to the putatively holistic perceptual process employed during facial recognition that does not require such dispersed visual sampling.

Using face stimuli to investigate the relationship in gaze dynamics between visual encoding and recognition has advantages over using most other stimulus categories. The properties and locations of important features are not nearly as heterogeneous across face exemplars as they are for most object or scene stimuli. Further, recent research has revealed that scan sequences during the recognition of faces are highly consistent across face exemplars and that such stereotyped gaze dynamics functionally relate to facial identification since higher scan sequence consistency has been correlated with higher facial identification performance [29]. Therefore, when using face stimuli, it is possible to spatially align data across experimental trials to achieve strong statistical power and interpretability in the contrast between encoding and recognition gaze dynamics. Due to this tractability and interpretability, gaining certain insights into visual encoding and recognition more generally may thus be uniquely possible with faces.

If the scan path replay hypothesis is instantiated in facial identity encoding and recognition, then eye movements might be expected to be identical between encoding and recognition of faces, particularly at the second fixation, given its functional importance. Further, such a hypothesis would imply that two fixations should also suffice for optimal face encoding, given that two fixations are all that are needed for optimal recognition. However, some limited evidence against this scan path replay hypothesis for faces comes from data incidentally reported in the aforementioned study by Hsiao and colleagues (2008) [2]. Data on the first two fixations that they report in a table suggest that the spatiotemporal dynamics of early fixation sequences differed between encoding and recognition. Specifically, those data suggest that average fixation location for the second ordinal fixation was lower on the face and fixation duration for the first ordinal fixation was longer during the test than the study phase (these reported differences are more than twice the standard errors, hence are putatively statistically significant). Notably though, an important potential confound in that study was the highly restricted viewing times during the test phase compared to the long stimulus presentation times during the study phase. Though the reported pattern of eye movements during the test phase was also seen for the unrestricted fixation condition, given that such trials were unpredictably interleaved among trials of restricted fixation, participants would likely still have had an expectation of restricted stimulus viewing time even during the unrestricted fixation trials. Only a few studies have examined the influence of temporal constraints within this range of durations on eye movements over faces [3,4,12,30], and differences in tasks and analyses in those studies make them difficult to relate to results of Hsiao and colleagues [2]. 

Consistent with this apparent difference in eye-movement patterns between study and test phase are some results incidentally reported in two investigations of facial recognition [10,31]. It must be noted though that both studies drew from the same dataset and contained the same limitation concerning temporal constraint differences between phases. One of these studies [10] reported in a supplementary analysis that, at the group level, participants made significantly fewer fixations to the left eye area and significantly more fixations to the nose area in the test compared to study phase during the second and third ordinal fixations. The other study [31], in part, investigated the influence of experimental conditions on individual differences in eye-movements. It reported that individuals’ eye-movement patterns within an early time window (i.e., the first second of viewing) were significantly modulated between study and test phase. Specifically, the degree to which individuals’ patterns were discriminable between each phase was significantly lower from the degrees individuals’ patterns were discriminable within each phase. Similar to the study of Hsiao and colleagues [2] though, there were differences in temporal constraints in viewing times between the study (up to 10 s) versus test (up to 1 s) phase. Therefore, it is unclear whether all these reported differences in eye movements between the study and test phases truly reflect differences between encoding and recognition processes or, rather, between unrestricted and restricted viewing times.

The present study was, thus, designed to distinguish between the influences of experimental phase (encoding/recognition) and of stimulus presentation time (short/long) on eye movement dynamics to faces. While having eye movements and behavioral performance (i.e., discrimination, response bias, and reaction times) measured, participants completed an encoding (“study”) phase and a subsequent recognition (“test”) phase, during which faces were judged to be old (i.e., presented in the study phase) or new. Each phase was divided into separate blocks of either one- or five-second stimulus presentation times so that participants knew how long the face stimulus could be expected to remain visible. Because of the varying number of fixations across trials and, importantly, because of the putative functional sufficiency of the first two ordinal fixations for optimal recognition [2], our eye movement dynamics analyses focused on the first two fixations. We found that old/new recognition performance increased for the long compared to the short study phase stimulus presentation time, indicating that, unlike for recognition, two fixations do not suffice for optimal face encoding. We further found influences of experimental phase on the temporal and spatial dynamics of eye movements within the first two fixations, demonstrating that eye movements are not replayed identically between encoding and recognition. The precise pattern of eye-movement dynamics subtly interacted somewhat with stimulus presentation time, though, indicating that the expectation of time constraint on stimulus viewing also affects the spatial pattern of eye movements. Because of these functional and dynamical differences that we observed between encoding and recognition, our results are inconsistent with a scan path replay hypothesis. Rather our results suggest that facial feature information is integrated over many fixations during encoding in order to form a robust unitized representation that can be rapidly and holistically activated during recognition within a small number of fixations.

## 2. Materials and Methods

### 2.1. Ethics Statement

Our protocol (#15-03683-XP) was approved by the Institutional Review Board of the University of Tennessee Health Science Center (since February 20, 2015). The study was carried out in accordance with the Code of Ethics of the World Medical Association (Declaration of Helsinki), and all participants gave written informed consent and were compensated for their participation.

### 2.2. Participants

We recruited 37 participants, all with normal or corrected to normal vision, for the study, but data from six participants were excluded because of poor eye-tracking calibration (4 participants) and because of concern about the degree of participant movement during data collection (2 participants). Thus, data from 31 participants (15 male; 25 right-handed) aged 20–44 years (mean 28.3, standard deviation 6.8 years) were analyzed.

### 2.3. Eye-Tracking

We used an EyeLink II head mounted eye-tracker (SR Research, Mississauga, ON, Canada), and sampled pupil centroid at 250 Hz during the trials of the experiment. Participants’ eyes were 57 cm from the stimulus display screen. The default nine-point standard EyeLink^®^ calibration was performed for each participant at the start of each experimental session, and a validation sequence was also performed before each of the six experimental blocks (24 trials per block). Both eyes were calibrated and validated, but only the eye with the lowest average maximum error was recorded for the trials following a particular calibration. Calibration was repeated when maximum error at validation was more than 1.33° of visual angle. Average validation error was always substantially lower than 1° of visual angle. The mean of the average validation errors was 0.36° of visual angle with a standard deviation of 0.10°. The mean of the maximum validation errors was 0.79° of visual angle with a standard deviation of 0.19°. To minimize head motion artifacts, all participants were seated on a stabilized drum stool with a back support, and had their heads fixed with a chin rest. Additionally, the “Head Camera” feature of the EyeLink II was engaged so as to provide some compensation for head motion that might still occur. Further, before each trial, a drift correction was performed. Saccade sensitivity was set to “Normal” (i.e., 30°/s velocity threshold and 8000°/s^2^ acceleration threshold), link/analogue filter was set to “standard”, tracking mode was set to “pupil”, and file sample filter was set to “extra”.

### 2.4. Stimuli

Ninety-six Caucasian-American (48 male) grayscale neutral expression frontal-view face images were used. The face images were all taken from the neutral expression 18 to 29-year-old age group of the Productive Aging Lab Face Database established by the University of Texas at Dallas (“Face Database-Park Aging Mind Laboratory”. Available online: http://agingmind.utdallas.edu/download-stimuli/face-database/ (accessed 1 January 2019)) [32]. Each face was scaled to have a forehead width subtending 10 degrees of visual angle at presentation and was rotated to correct for any tilt of the head. Images were cropped to remove most of the background, but not the hair or other external features, and all face images were equated for overall luminance. We chose not to remove the external facial features from our stimuli, as has been done in some other studies, because whole head stimuli are more ecological compared to stimuli isolating the internal facial features and because very few fixations are directed to the external features even when they are present (e.g., [33]). At presentation, images were centered on a black background. To eliminate any possible stimulus bias as the source of any laterality effects, half of the faces were randomly left-right flipped across the vertical midline of the image for each participant. The website of the Productive Aging Lab Face Database states: “This [database] contains a range of face of all ages which are suitable for use as stimuli in face processing studies. Releases have been signed by the participants we photographed and the faces may be included in publications or in media events”.

### 2.5. Design and Procedure

**Figure 2:**
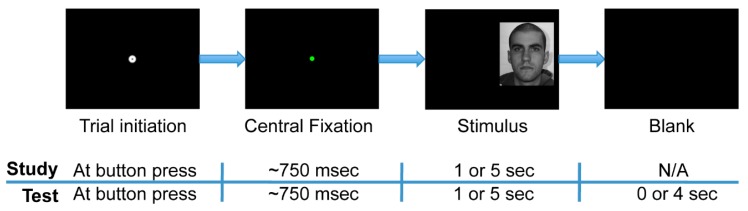
Schematic of the experimental trial sequences. Participants initiated trials with a button press, and following a brief and enforced central fixation, the facial stimulus appeared to the left or right. In Study phase trials, each facial stimulus was displayed for either one or five seconds total, depending on the block. In the Test phase, facial stimuli appeared for up to either 1 or 5 s, depending on the block, and within five seconds of stimulus onset, participants were required to respond whether the face had been in the Study phase or not (i.e., whether the face was “old” or “new”).

The experiment was comprised of two phases: study and test (Figure 2). Further, each phase was divided into separate experimental blocks within which face stimulus presentation times were either short or long. During the study phase, participants observed a series of 48 faces (24 female), such that one face was presented per trial. Participants were instructed to study the faces so as to recognize them in the test phase. The study phase was split into two experimental blocks of 24 trials each. In one of the two blocks, all of the face stimuli were presented for one second (“short” presentation), and in the other block, all were presented for five seconds (“long” presentation). In the study phase, a trial terminated only once the full presentation time had elapsed. The one-second stimulus presentation time condition typically allowed for 2 to 3 uninterrupted fixations to each face (mean 2.41, standard deviation 0.49 uninterrupted fixations), and so such a time window was comparable to the restricted fixation conditions in the experiment of Hsiao and colleagues (2008) [2].

The test phase immediately followed the study phase. During the test phase, participants observed a series of 96 faces comprised of the original 48 study phase (“old”) faces plus 48 new faces. Participants indicated with a button press whether or not they recognized each stimulus as one observed during the study phase (old/new task). Participants were instructed to respond as soon as they thought they knew the answer and to guess when they were not sure. The test phase was divided into four experimental blocks of 24 trials each. Each block contained 12 “old” and 12 “new” faces presented in a pseudorandom order. One-second stimulus presentation time limits existed in two of these blocks, and five-second limits existed in the other two blocks. Furthermore, all of the “old” faces in one of the two short-presentation blocks in the test phase were faces that had had short presentations in the study phase, while those in the other test phase short-presentation block had had long presentations in the study phase. This property likewise held for the two long-presentation blocks in the test phase. For all test phase trials, participants were given up to five seconds following stimulus onset to respond, regardless of the presentation time limit of the stimuli. The trial ended immediately upon response, so the one- and five-second stimulus presentation limits within the test phase were only upper limits, not enforced viewing times.

The order of the short and long stimulus presentation blocks within the study phase was counterbalanced across participants. The “old” faces in the first two blocks of the test phase were those faces contained within the first block of the study phase, and likewise the “old” faces in the last two blocks of the test phase were those contained within the second block of the study phase. Within the test phase, the short and long stimulus presentation blocks alternated and their order across participants was counterbalanced between the two possible orders for the study phase blocks. Thus, with respect to short and long stimulus presentation time, there were four possible combinations of study and test phase block orders (Table 1).

The participants initiated each trial of the experiment in a self-paced manner. Before stimulus onset, participants fixated the start position at the center of the screen, indicated by a standard Eyelink II calibration target (0.17° diameter black circle overlaid on a 0.75° diameter white circle) on the black screen. Participants initiated the trial by pressing a button while looking at the fixation target. In this action, a drift correction was performed. A colored dot (0.05° diameter) remained after drift correction, and the stimulus appeared only after the participant had fixated the dot for an accumulated total of 750 ms. This process ensured that drift correction and fixation were stable prior to stimulus onset. If more than 750 ms of fixation away from the start position accumulated before the trial could be initiated, drift correction was repeated. A fixation was considered to be off the start position if it landed more than 0.5° from the center of the dot. Dot color changed successively from red to yellow to green in order to signal to the participant that a maintained fixation was successfully detected at the start position.

Because fixation patterns are affected by visuo-motor factors such as left/right pre-stimulus start position [33,34], and not just stimulus factors such as facial physiognomy [10], we counterbalanced the side of the screen (i.e., left or right) that the face appeared relative to the central fixation dot at the beginning of each trial. We, thereby, counterbalanced the pre-stimulus start position relative to the face to control for visuo-motor influences on eye movement patterns. Position along the y-axis of the screen was calculated uniquely for each face stimulus such that the central starting fixation dot would always have the same y-coordinate component as the unique point equidistant from all of the nearest internal facial features. Specifically, that unique coordinate was calculated numerically for each face such that it was equidistant from the centers of the nearest eye, nearest half-nose, and nearest half-mouth regions that had been manually designated for this purpose. Distance from the central starting fixation dot to the midline of the face was always 8 degrees of visual angle along the *x*-axis.

The order of the stimuli was pseudo-randomized such that within each phase, there were equal proportions of trials for each combination of levels of the factors of stimulus presentation time limit, start position, and face gender. The particular subset of faces used in the study phases was randomized across participants. Of the faces presented in both the study and test phase, all were presented on the same side of the visual field at study and test. The experiment was programmed in Python and interfaced with the eye-tracker using the PyLink libraries.

It is worth noting a few aspects of our experimental design that differed from those of Hsaio and Cottrell (2008) [2]. All of these differences served to make our design more ecological and, thus, enable our findings to be more generalizable to facial recognition processes that are common in daily life. First, our presentation of left- and right-appearing stimuli differs from the design of Hsaio & Cottrell (2008), in which stimuli were presented above and below the initial fixation. In typical daily visual experience, lateral saccades are more common than are vertical saccades [35]. Further, having starting fixation locations that are lateral to the faces, as opposed to above and below the faces, afforded us greater control over how distant participants’ gaze started off relative to all of the internal facial features. Second, our stimuli were not forward or backwards masked as in Hsaio and Cottrell (2008), since, in real life, faces are not usually masked before or after we look to them and because the facial information processed in peripheral vision before the first saccade to a face may be important to the subsequent visual processing and eye-movement dynamics.

### 2.6. Analyses

#### 2.6.1. Behavior

We assessed participants’ discrimination performances, response biases, and reaction times during the old/new recognition task of the test phase. Specifically, *d’* (*z*(hit rate) − *z*(false alarm rate)) and criterion *c* (−[*z*(hit rate) + *z*(false alarm rate)]/2) were computed for each combination of the study and test phase presentation time conditions for each participant. Because rates at ceiling or floor (i.e., 100% or 0%, respectively) produce infinite values for these signal detection measures, we applied the Goodman correction [36,37] to preclude this artefact. Study phase presentation time for an “old” face in the test phase was defined by how long the same face image had been presented in the study phase. Note that because “new” faces in the test phase did not correspond to either of the study phase stimulus presentation time conditions, a given false alarm rate was calculated using just the “new” trials within the same experimental block from which the corresponding hit rate was calculated. For each study and test phase time condition, reaction times were analyzed for correct trials only. Reaction times were calculated only for “old” faces because, again, “new” faces in the test phase did not belong to either of the study phase stimulus presentation time conditions. Additionally, median, rather than mean, reaction times were calculated for each participant (as is common practice for reaction time analyses) because reaction time distributions tend to be skewed to high reaction times [38,39,40,41,42] and, thus, simply using median as a measure of central tendency is good practice under typical experimental circumstance [38,39,40,41,42], unless, for example, sample sizes differ [43] or are small [44]. The mean reaction times displayed in our figure are the means of the participant medians.

#### 2.6.2. Eye Movement Pattern Analyses Overview

Because area of interest (AOI) analyses can be criticized for requiring a highly subjective a priori segmentation of visual features [45], while spatial statistical maps can be criticized for lacking statistical sensitivity [10], we conducted analyses that would allow for good statistical contrast sensitivity without the need for subjective segmentation. In particular, we calculated vertical-profile fixation densities, which can visualize fixation density over specific facial features (eyes, nose, mouth) without respect to laterality or fine differences in horizontal position. We then mapped statistical differences in vertical-profile density between conditions by performing a Monte Carlo permutation test that was then corrected for false discovery rate (FDR). Only the first two ordinal fixations were analyzed because of the variable number of fixations between stimulus presentation time conditions and because of prior research revealing that the first two fixations are functionally sufficient during face recognition [2]. Because the recognition performance results we report are indeed consistent with the functional sufficiency of the first two fixations at recognition, this analytic constraint, therefore, conveniently corresponds to those fixations most functionally relevant to our participants during facial recognition. Additional details about these eye movement analyses are contained within the following paragraphs.

#### 2.6.3. Analysis Software

Eye movement data were obtained through EyeLink Data Viewer software by SR Research. Subsequent analyses on these data and on the behavioral data from the test phase were performed with custom Matlab (The MathWorks, Inc., Natick, MA, USA) code. Some statistical tests were also performed in SPSS (IBM, Somers, NY, USA).

#### 2.6.4. Profile Density Analyses

Vertical-profile densities were the result of summing along the horizontal dimension (*x*-axis) of two-dimensional spatial density heatmaps in which fixations were plotted as Gaussian densities with a standard deviation of 0.26° of visual angle in both the x and y dimensions. Because each fixation was plotted with equal density and spatial extent, individual fixations were thus not weighted by their durations.

#### 2.6.5. Profile Density Statistical Contrast Analyses

In order to produce maps of statistically significant differences in the profile density map contrasts, a Monte Carlo permutation test was performed on fixation locations between the contrasted conditions. A Monte Carlo permutation test (also called an approximate permutation test or a random permutation test) is a standard, accurate and robust method of performing a significance test on data that is not known to have a parametric (e.g., normal) distribution of values, such as our data. This type of statistical analysis method has been applied to eye-tracking data in previous studies [10,33,46] and is based on methods applied in the analysis of functional brain imaging data [47]. Use of profile density statistical analyses such as those in the current study has been motivated in detail in a prior eye-tracking study of face perception [10]. 

The null hypothesis in the Monte Carlo permutation tests was that the distributions of fixation locations for each ordinal fixation (i.e., fixation 1, fixation 2) were the same between the contrasted conditions (e.g., study phase long presentation versus test phase short presentation). Thirty-nine thousand resampling iterations were performed for each statistical map. For each iteration, the two-dimensional locations of fixations were resampled for each individual participant according to the assumed exchangeability criteria that corresponded to the null hypothesis for the given contrast (i.e., that fixation locations were exchangeable between the two contrasted conditions). Then a new resampled 2-dimensional spatial density contrast was produced. These resampled maps were then averaged across participants to produce 39,000 group difference maps, the distribution of which was used to determine statistical significance.

To find regions of statistically significant difference in vertical-profile density, the resampled iterations from the relevant spatial density Monte Carlo permutation test were summed along the horizontal dimension to produce the resampled iterations of a vertical-profile Monte Carlo permutation test. *p*-Values were computed pixel-wise (i.e., at each pixel along the y-dimension) based on the number of corresponding pixels in the resampling iterations that were greater than a given positively valued pixel (i.e., where condition 1 had a greater profile density) in the true profile density difference and that were less than a given negatively valued pixel (i.e., condition 2 greater) in the true profile density difference. False discovery rate (FDR) correction was then applied to these profile density statistical contrasts. Plots indicate statistically significant differences at a threshold of *q* < 0.05, which corresponds to an estimated false discovery rate of 5% among the profile coordinates designated as statistically significant. FDR control took into account all pixels across all the maps of a given contrast type (e.g., for short versus long presentation time contrasts, a single correction was performed including both the study and test phase maps). In these maps visualizing significant differences, pixels along the entire orthogonal dimension of the average face image were highlighted where the dimension of interest had a significantly different profile density between contrasted conditions.

## 3. Results

### 3.1. Task Performance Measures

#### 3.1.1. Discrimination Reduced for Short Study Time

**Figure 3:**
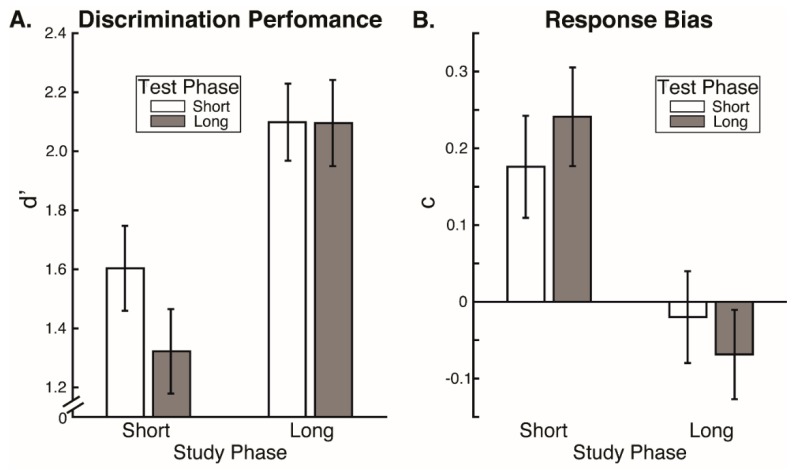
Recognition performance. (**A**) Discrimination performance was reduced for faces that had been studied for only one second compared to those that had been studied for five seconds and (**B**) Response bias indicated that the short study phase stimulus presentation time elicited more conservative criteria to report that a face was recognized than did long study phase stimulus presentation time.

Discrimination performance was reduced for faces that had been studied for only one second compared to those that had been studied for five seconds. The two-way ANOVA on discrimination (d’) scores (Figure 3A), with study phase stimulus presentation time (one second, five seconds) and test phase stimulus presentation time limit (one second, five seconds) as within-subject factors revealed a significant main effect of study phase stimulus presentation time (F(1,30) = 30.00, *p* = 0.0000061, η_p_^2^ = 0.50), such that discrimination performance scores were lower for short compared to long presentation time (mean difference: 0.67). There was no significant main effect of test phase stimulus presentation time limit (F(1,30) = 1.36, *p* = 0. 25, η_p_^2^ = 0.043), nor was there a significant interaction between the study and test presentation time conditions (F(1,30) = 1.57, *p* = 0.22, η_p_^2^ = 0.050).

#### 3.1.2. Conservative Criterion for Short Study Time

Criterion c scores estimated bias in responding that a face was recognized, where a higher criterion score indicates a stricter criterion (i.e., more reluctance when uncertain to respond that a face was recognized). The two-way ANOVA on criterion scores (Figure 3B), with study phase stimulus presentation time and test phase stimulus presentation time limit as within-subject factors revealed a significant main effect of study phase stimulus presentation time (F(1,30) = 18.23, *p* = 0.00018, η_p_^2^ = 0.38), such that criterion scores were higher for short compared to long study phase presentation time (mean difference: 0.25). There was no main effect or interaction involving test phase stimulus presentation time limit (both F(1,30) < 1.12, *p* > 0.29, η_p_^2^ < 0.036). One-sample *t*-tests on criterion scores for each of the four study phase by test phase stimulus presentation time condition combinations further revealed that scores for short stimulus presentation time in the study phase significantly differed from zero for both of the test phase stimulus presentation time limits (both t(30) > 2.59, *p* < 0.015, two-tailed, d_Cohen’s_ > 0.46). The remaining criterion values did not significantly differ from zero (both t(30) < 1.16, *p* > 0.25, two-tailed, bias-corrected d_Cohen’s_ < 0.21). These results reveal that short study phase stimulus presentation time elicited more conservative criteria to report that a face was recognized than did long study phase stimulus presentation time. Those higher criteria were also more conservative than that of the ideal observer (i.e., C = 0, where the probability of misses and false alarms are conjointly minimized, given the available information and the uncertainty), and were, thus, not optimal criteria, given the parameters of our experiment. In the foregoing d’ and criterion analyses, 12.1% of participants’ calculations (out of all 124: 4 for each of the 31 participants) required an adjustment of hit rate from ceiling, 12.1% required an adjustment of false alarm rate from floor, and 4.8% required both adjustments (see Materials and Methods, Section 2.6.1.).

#### 3.1.3. Reaction Time

The two-way ANOVA for reaction time, with study phase stimulus presentation time and test phase stimulus presentation time limit as within-subject factors, did not reveal significant main effects or interactions (all F(1,30) < 1.47, *p* > 0.235, η_p_^2^ < 0.047). Pooling together of all presentation time conditions revealed that overall mean reaction time was 1459 ms (standard deviation 384 ms).

### 3.2. Temporal Dynamics of Fixations

#### 3.2.1. Latencies to First Saccade

**Figure 4:**
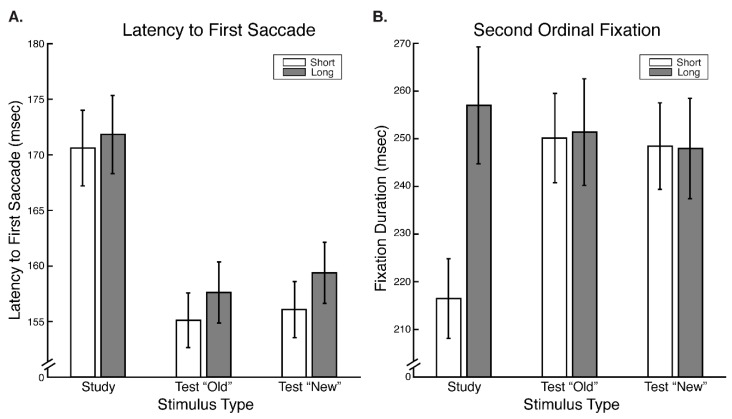
Temporal dynamics of eye-movements. (**A**) Latency to first saccade and (**B**) fixation durations for the second ordinal fixation. Each is plotted by stimulus experience type (Study, “old” test face, “new” test face) and stimulus presentation time condition (white bars = “short” one second, gray bars = “long” five seconds). Latency to first saccade was longer (*p* < 0.0005) during the study than the test phase. Fixation duration for the second ordinal fixation was shorter (*p* < 0.005) for the short study phase condition than for other conditions.

Latencies to first saccade were longer during the study phase compared to the test phase. A two-way ANOVA was conducted on participants’ median latency to first saccade (Figure 4A), with stimulus experience category (study phase face, test phase “old” face, test phase “new” face) and stimulus presentation time condition (one second, five seconds) as within-subject factors. There was a significant main effect of stimulus experience category (F(1.26,33.86) = 47.97, *p* < 0.0005, Greenhouse–Geisser corrected (ε = 0.63), η_p_^2^ = 0.64), but no main effect or interaction involving stimulus presentation time (both *p* > 0.10, η_p_^2^ < 0.093). Paired *t*-tests among stimulus experience categories on participants’ median latencies (with stimulus presentation time conditions pooled together) revealed that latencies to study phase faces were longer than to both the “old” and “new” test phase faces (both t(28) > 5.94, *p* < 0.0005, two-tailed, bias-corrected G_Hedges_ > 0.74). Latencies to “old” and “new” test phase faces did not differ (both t(29) = 0.49, *p* = 0.63, two-tailed, bias-corrected G_Hedges_ = 0.031). Three participants had outlier data (i.e., ±2.5 standard deviations from the group median) in at least one condition, and so were excluded from the ANOVA and from those paired *t*-tests involving the condition(s) in which their data were outliers. Inclusion of these outlier data points, however, do not change the pattern of results.

**Figure 5:**
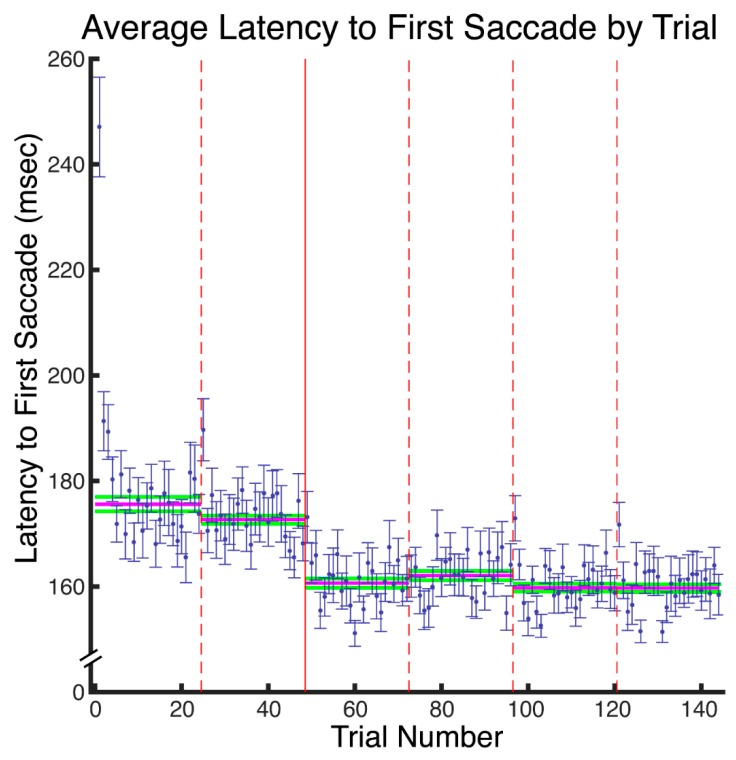
Latency to first saccade by trial into experiment. The average of the participants’ median latencies to first saccade are plotted as a function of trial number. The blue error bars indicate standard errors of the mean. Vertical red lines delineate the blocks of the experiment and the solid red line specifically delineates the study and test phases of the experiment. Magenta horizontal lines indicate the block means of these average trial latencies and the green horizontal lines indicate their respective standard errors. Because the first trial of each block tended to exhibit much longer latencies than other trials, probably related to needing to become engaged with the task, the first trial of each block was excluded from the block averages. This analysis indicates that latencies to first saccade did not gradually become shorter as a function of the number of trials into the experiment, rather there was a clear step-wise shortening of latencies from the study phase to the test phase of the experiment.

It is conceivable that the longer median latencies to first saccade during the study phase compared to the test phase merely reflect a gradual shortening of latencies as a function of the number of trials into the experiment, rather than of the experimental phase as such. However, we found no evidence that latencies gradually shortened throughout the experiment. Rather we observed a clear step-wise shortening of latencies from the study phase to the test phase (Figure 5).

#### 3.2.2. Fixation Durations

We found no differences in participants’ median fixation durations between correct and incorrect trials. Thus, we have included all of the trials in our final fixation duration analyses, so as to maintain high statistical power by not reducing the number of trials going into our analyses more than was necessary. Specifically, all paired comparisons between hits and misses (i.e., between correct and incorrect “old” face test phase trials) and between correct rejections and false alarms (i.e., between correct and incorrect “new” face test phase trials) across both stimulus presentation time conditions (one and five seconds) and across both of the first two ordinal fixations failed to yield any statistically significant differences (all eight comparisons *p* > 0.092, two-tailed, uncorrected).

There was a trend for fixation durations of the first ordinal fixation to be shorter during the study phase than during the test phase. For the first ordinal fixation, a two-way ANOVA was conducted on participants’ median fixation durations (Supplementary Figure S1), with stimulus experience category (study phase face, test phase “old” face, test phase “new” face) and stimulus presentation time condition (one second, five seconds) as within-subject factors. One participant with outlier data in some conditions was excluded from this ANOVA, though inclusion of that participant does not change the pattern of results. Though stimulus experience category suggested a trend (F(1.15,33.47) = 3.36, *p* = 0.07, Greenhouse–Geisser corrected (ε = 0.58), η_p_^2^ = 0.104), there was no main effect or interaction involving stimulus presentation time condition (both *p* > 0.20, η_p_^2^ < 0.054). This apparent trend for stimulus category reflects study phase fixation durations being numerically shorter than either of the “old” or “new” face test phase conditions.

Fixation durations of the second ordinal fixation were significantly shorter during the short study phase condition than during any of the other conditions (Figure 4B). For the second ordinal fixation, the two-way ANOVA on participants’ median fixation durations with stimulus experience category (study phase face, test phase “old” face, test phase “new” face) and stimulus presentation time condition (one second, five seconds) as within-subject factors, yielded a significant interaction (F(2,52) = 6.88, *p* = 0.0022, η_p_^2^ = 0.209). Paired *t*-tests revealed that this interaction was driven by shorter fixation durations for the short study phase than for all other conditions (all were *p* < 0.005, two-tailed, bias-corrected G_Hedges_ > 0.57). There were no differences among the other conditions (all were *p* > 0.49, two-tailed, bias-corrected G_Hedges_ < 0.14). Four participants had outlier data in at least one condition, and so were excluded from the ANOVA and from those paired *t*-tests involving the condition(s) in which their data were outliers, although inclusion of these data points did not change the pattern of results. Also, if a second ordinal fixation spanned the offset of the stimulus, whether due to the stimulus presentation time limit or to trial termination following from a response made by the participant, that fixation was excluded from the fixation duration analyses so that the analyzed fixation durations would only reflect those of uninterrupted fixations. On this basis, 12.5% of one-second study, 13.7% of one-second test, 0% of five-second study, and 3.4% of five-second test condition fixations were excluded from the fixation duration analyses. Finally, incorrect trials in the test phase were not excluded from our analyses since we found no significant differences in fixation duration among hit, false alarm, correct rejection, or miss trials (Supplementary Results). 

### 3.3. Spatial Patterns of Fixations

Vertical-profile density statistical contrasts between the “old” and “new” face trials of the test phase did not reveal any significant differences in either the first or second ordinal fixation for any contrasts of presentation time condition. For this reason and to restrict the number of statistical tests conducted, subsequent profile density statistical contrasts involving test phase trials were conducted pooling fixation data from “old” and “new” test phase trials. Note that when this involved comparing data of different sample sizes between phase conditions (i.e., 48 study face trials versus 96 test phase trials), average densities for the test phase were scaled by ½ to be comparable with the study phase densities.

In the first ordinal fixation, vertical-profile density statistical contrasts revealed only a small lower eye region of relatively greater density in the test than study phase trials of the long presentation time condition, but revealed no differences between the study and test phases of the short presentation time condition (Supplementary Figure S2A). Two-dimensional analyses of the same contrast were consistent with this (Supplementary Figure S3). There were also no significant differences between the short and long stimulus presentation time condition trials for either phase (Supplementary Figure S4).

**Figure 6:**
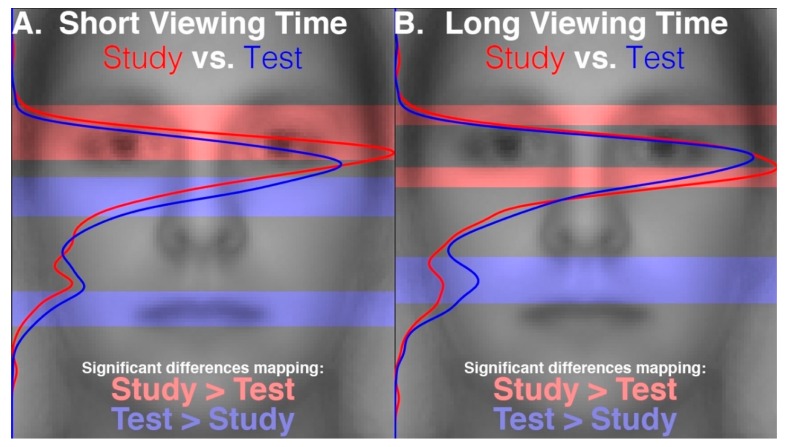
Study versus test phase vertical-profile density curves and statistical contrasts for the second ordinal fixation, separated by (**A**) the short presentation time condition; and (**B**) the long presentation time condition. The face image is highlighted where relative vertical density was significantly different (q < 0.05) between the contrasted conditions. Curves are scaled relative to the maximum density between the two given contrasted curves. The densities are only interpretable relative to one another and are dimensionless quantities; therefore, no units or absolute values are reported.

In the second ordinal fixation, no differences were detected between short and long stimulus presentation time conditions for either the study or test phases (Supplementary Figure S5). This suggests that there was no main effect of stimulus presentation time on the spatial pattern of fixations during the second ordinal fixation. However, there were significant differences between the study and test phase trials for both the short and long stimulus presentation time conditions (Figure 6). Specifically, there was relatively greater fixation density over the eye region for study phase trials than for test phase trials. Further, there was greater fixation density over lower facial features for test than study phase trials. This suggests that, at least at a coarse level, a main effect of phase was present such that study phase attracted relatively greater eye region fixation and test phase attracted greater fixation over lower facial features. The precise pattern of differential fixation density between the study and test phases differed between short and long stimulus presentation time conditions, though, suggesting that there was an interaction between phase and stimulus presentation time conditions on fixation density in the second ordinal fixation. Two-dimensional analyses of the same contrast are also consistent with this (Supplementary Figure S6).

To further characterize the interaction between phase and stimulus presentation time condition, vertical-profile density statistical contrasts for the second ordinal fixation were conducted between the study phase short presentation and test phase long presentation conditions as well as between the study phase long presentation and test phase short presentation conditions (Supplementary Figure S7). This again revealed the coarse main effect of phase, with the study phase containing relatively greater eye region fixation and the test phase containing relatively greater fixation over lower facial features. An interaction between phase and time was again evident from the variation in the precise pattern of differential profile density between both the study and test phase contrasts.

It should be emphasized that the significant differences in profile density between study and test phases that we detected in the second ordinal fixation are *relative* differences. Plots of vertical-profile density (Figure 6) for the second ordinal fixation indicate that absolute fixation density was greatest over the lower eye region for all conditions, notwithstanding the relative differences among the conditions. From these plots, it is further evident that the magnitudes of the significant relative differences are small. However, an additional exploratory analysis testing whether the consistency in ordinal fixation locations between study and test are functionally related to facial recognition performance yielded no significant correlation for any condition (all *p* > 0.21, Supplementary Figure S8). This provides further evidence against the scan path replay hypothesis.

For completeness, we also returned to the first ordinal fixation and compared the vertical-profile density between the study phase short presentation and test phase long presentation conditions as well as between the study phase long presentation and test phase short presentation conditions. This comparison yielded no significant differences (Supplementary Figure S9). This result verified that, in addition to there being no significant main effects of phase and stimulus presentation time condition on the fixation patterns of the first ordinal fixation as described above, there were also no significant interactions between these factors on the fixation patterns of the first ordinal fixation. This result contrasts with the second ordinal fixation, which, as detailed above, did show significant effects involving these factors.

Finally, although the scope of our study concerns only the first two ordinal fixations and participants did not always make three fixations in the short presentation time conditions, we also conducted an exploratory analysis on what data was available for the third ordinal fixation (Supplementary Figure S10). This analysis did not indicate any robust effects.

### 3.4. Areas of Interest Analysis

In the introduction, we discussed the possibility that if time window is not controlled for in analyses contrasting eye movement patterns between study and test phase, spurious differences could be introduced. To illustrate this, we compared five seconds of gaze data collected during facial encoding to the same data truncated to one second, and we found robust and significant differences in the relative proportions of viewing time at the eyes and nose that match the pattern of differences reported by Henderson and colleagues [28] as mentioned in the Introduction (Figure 1). This constitutes a clear empirical confirmation and exemplification of an analysis-dependent artefact that can be attributed entirely to the difference in analysis time window length, given that the comparison is of gaze data that is not even statistically independent (i.e., the one and five second data come from the same sample, thus, compared to independent data, there should theoretically be a bias against finding statistical differences).

Rectangular areas of interest (AOIs) were manually drawn uniquely for each face around the right and left eyes, bridge of nose (i.e., middle of eye region), nose, and mouth as determined by identical drawing criteria to those described in Arizpe et al. (2015) [34]. These AOIs were never visible to participants during the experiment and were for analysis only. To form comparable AOIs to those utilized in Henderson et al. (2005) [28], our left eye, bridge, and right eye AOIs were combined into one “eyes” AOI.

The mean proportion of total gaze dwell time in each AOI across the trials of the five second study phase condition was calculated for each participant. For each AOI, we conducted paired *t*-tests between participants’ data from the entire five seconds of the trials and from the same data that had been truncated to include only the first second of the trials. These two analysis time windows produced highly significant differences in proportion of dwell time on the eyes (t(30) = −4.06, *p* = 0.0003, two-tailed), nose (t(30) = −4.95, *p* = 0.000027, two-tailed), and “other” AOIs (t(30) = 12.90, *p* = 9 × 10^−14^, two-tailed). The difference in the mouth AOI was not statistically different, but showed a trend (t(30) = 1.70, *p* = 0.10, two-tailed). The pattern and magnitude of the differences match those reported by Henderson and colleagues (2005) [28] as between the encoding and recognition phases of their experiment. Thus, we provide evidence that the pattern of differences in gaze that were proposed by Henderson and colleagues [28] as being the differences between encoding and recognition may, instead, largely, or even entirely, be artefacts of the differences in analysis time window that were applied between the two phases of their experiment.

## 4. Discussion

Our results reveal that eye movement dynamics differ between encoding and recognition of faces and that longer sequences of eye-movements are functionally necessary to achieve optimal encoding than are necessary to achieve optimal recognition. Within the first two fixations, we found differences in the temporal and spatial dynamics of eye movements between encoding and recognition. For the study compared to the test phase, we found significantly longer latencies to first saccade and relatively greater fixation density over the eyes along with relatively less fixation density over the lower facial regions during the second ordinal fixation. We also found evidence, though, that stimulus presentation time and experimental phase interacted somewhat in the dynamics for these early eye movements. In particular, fixation duration of the second ordinal fixation was shorter in the one-second study phase condition compared to other conditions (i.e., compared to five-second study, one-second test, and five-second study conditions). Also, though the coarse-level fixation density differential between upper and lower facial features held regardless of the presentation time condition, the fine-grained pattern of differential fixation density was not identical across stimulus presentation time conditions. Most importantly, the long versus short study phase presentation time conditions caused improved recognition performance, whereas the long versus short test phase conditions did not, demonstrating that optimal encoding is not achieved as rapidly as is optimal recognition.

These results are consistent with and explain the study versus test phase eye movement differences that could be inferred from data incidentally reported by Hsiao and Cottrell (2008) [2]. In a table, they reported (at least numerically) that average fixation location for the second ordinal fixation was lower on the face and that duration for the first ordinal fixation was longer during test than study phase. Restricted stimulus presentation time during the test phase was a potential confound though. Our results imply that these apparent effects were indeed due to the differences in cognitive processing between encoding and recognition, rather than due to differences in stimulus viewing time constraints between the study and test phase. The relatively greater fixation density over lower versus upper facial regions during recognition compared to encoding that we observe elucidates why Hsiao & Cottrell detected an average fixation location apparently lower on the face during the test phase compared to the study phase. Notably, though our average fixation location was lower on the face during recognition due to some shift of density toward lower facial features, absolute fixation density was still always greatest over the eye regions during both encoding and recognition. Finally, the trend for shorter durations of the first ordinal fixation during encoding compared to recognition that we observe (Supplementary Figure S1) also corresponds to the same pattern apparent in the results of Hsiao & Cottrell.

The test phase of our experiment contained faces that participants had previously seen in the study phase; however, facial novelty versus familiarity as such does not account for the eye movement differences we observe between the study and test phase. Several previous studies have reported that fixation patterns to faces differ between novel and familiar faces, with effects observed for faces that are familiar because they are famous [48,49,50,51], personally familiar [52,53], or even familiar simply from repeated recent exposure [54,55,56]. Thus, it is important to distinguish potential familiarity effects of previous exposure from effects of the encoding versus recognition processes being employed. Importantly though, we detected no eye movement differences between the “old” and “new” face test phase trials in the first two fixations. This null difference between “old” and “new” is consistent with prior studies, considering that effects of facial familiarity have been reported to appear in later rather than earlier ordinal fixations [52] and that most eye-tracking studies reporting facial familiarity effects pool more fixations than just the first two in the analyses. Further, robust familiarity effects have been reported to arise only after multiple exposures to a face [54]. Considering the evidence that only the first two ordinal fixations are sufficient for optimal facial recognition [2], it is possible that facial familiarity effects on eye movements are only present for later ordinal fixations that are functionally superfluous to the facial recognition process. Regardless, the eye movement differences that we observe between study and test phase within the first two ordinal fixations appear to be exclusively accounted for by differences between encoding and recognition processes and not by previous exposure to some of the faces.

### 4.1. A Novel Account of Encoding and Recognition

Our results are not consistent with a strict scan path replay hypothesis [15], under which the eye movement sequences employed during encoding are replayed identically to accomplish recognition. Our results indicate that fixations made during encoding are not replayed identically during recognition, but rather that there are systematic differences in eye movements between encoding and recognition phases. It must be noted, though, that absolute fixation density was greatest over the lower-eye region for all conditions. The relative differences were small in magnitude, and the functional significance is unclear. Considered alone then, our eye movement evidence leaves open the possibility for a more approximate scan path replay hypothesis, which might allow for some subtle differences between encoding and recognizing eye movement sequences.

However, more substantial evidence against even this possibility is our finding that eye movement sequences during encoding had to be longer than during recognition for optimal recognition performance, so could not be considered to be replayed sequences. We found no effects of test phase stimulus presentation time limits on recognition performance. This is consistent with prior research indicating that two fixations suffice for optimal face recognition [2,12]. If anything, there was a numerical trend of lower discrimination performance for the longer test phase presentation time limit, suggesting that more fixations beyond the second could even interfere with recognition performance. Importantly though, discrimination performance was higher for the long study phase presentation time condition compared to the short, and criterion response bias was more conservative than optimal for the short compared to the long study phase stimulus presentation time condition. Even in the short stimulus presentation time condition, participants were typically able to make at least two full fixations. Therefore, our results indicate that while two fixations may suffice for optimal recognition, they do not suffice for optimal encoding. A scan path replay mechanism would imply that the fixation sequence sufficient for recognition would also be sufficient for encoding; however, this is not the case.

Altogether, the evidence suggests a different and novel mechanism relating encoding and recognition. Specifically, encoding seems to entail an integration of disparate feature information across multiple fixations. This integration forms a robust unitized representation that can be activated rapidly and holistically at recognition within substantially fewer numbers of fixations. Prior research characterizing the distribution of multiple fixations during face encoding (e.g., [33,34,35,52,57]) reveals that beyond the second ordinal fixation, the distribution of fixations becomes less stereotyped and more spatially dispersed. Also, when fixation is spatially restricted during face encoding, recognition performance is decreased compared to when there is no restriction [28]. Thus, it is evident that optimal face encoding functionally requires a dispersed sampling of the specific visual features of a face through multiple fixations.

In contradistinction, recognition performance is optimal within two fixations, likely reflecting what is already widely supported within the face identification literature, namely, that a face identity representation previously encoded is activated through visual processing at recognition that is holistic in nature (i.e., processed as a unitized, non-decomposable whole; [58,59]). Indeed, observers tend to prefer to fixate at a featureless facial location between the eyes and nose that is visually optimal for such putative holistic processing [1]. Both recognition as well as some neural processing of facial features are tuned to visual field location within the retinotopic reference frame corresponding to such a preferred fixation location [60,61]. However, there is also evidence for individual differences in this tuning, both with respect to retinotopic location [8,30] and spatial frequency [62].

The results of two recent individual differences studies [63,64] have been interpreted in a way partially contradictory to the account of gradual feature integration at encoding for rapid holistic recognition that we have just proposed. However, both the analyses and the interpretation of those studies can be fundamentally criticized. In those studies, each participant’s eye-movements were modeled as Hidden Markov Models (HMMs) and those HMMs were partitioned into groups labeled as “holistic” and “analytic”. Participants whose HMMs were more similar to a representative “analytic” HMM had higher recognition performance. Thus, the results were interpreted as indicating that analytic, not holistic, eye movement patterns at recognition are associated with better recognition performance. Because our critique of these studies is somewhat technical, detailed discussion is contained in our Supplementary Materials. Briefly stated though, it is evident that that the properties of eye-movements fail to satisfy the assumptions of HMMs. Therefore, it is difficult to interpret the characteristics of resulting HMMs and any differences among HMMs. Further the number of the groups of HMMs was not discovered, but rather imposed a priori, and the labeling of these HMM groups as “holistic” and “analytic” is disputable. Indeed, the group(s) labeled as “analytic” had fixations mainly restricted to regions just below the eyes, a location optimal for rapid, and putatively holistic, facial recognition [1,12]. Thus, the group(s) labeled as “analytic” could rather be considered a holistic group. Additionally, the “holistic” group had fixations notably more widely dispersed, and so participants of the group typically foveated more facial features than participants of the other group(s). Thus, the group labeled as “holistic”, could rather be considered an analytic group. Given the association between individual differences in holistic processing and in face recognition ability [65,66,67], it would be expected that eye movement patterns optimal for holistic processing (i.e., more like the so called “analytic” group) would correlate with recognition performance. Thus, even when ignoring the analytic issues, the reported results of those two studies are consistent with this account of holistic recognition, though they have been interpreted otherwise.

For our experiment, we utilized identical images for the study phase stimuli and the corresponding test phase stimuli. A scan path replay hypothesis would predict that using the same images between encoding and recognition would enhance the replaying of eye-movements. Thus, this aspect of our design, theoretically, gives such predicted scan path recapitulation dynamics the highest likelihood of emerging. Also, the possibility of being able to confirm such recapitulations in our analyses were maximized, given that maps of eye-movements could be straightforwardly aligned. Additionally, the task instructions to expect to be tested on recognition for the images from the study phase would have created top-down influences more likely to lead to scan path recapitulation compared to if no instructions had been given. Strikingly, even given all these favorable conditions for observing scan path recapitulation dynamics, we did not find evidence in support of such recapitulation. In fact, the scan path replay hypothesis is problematic from a purely theoretical standpoint as a general theory of visual recognition in that it is ecologically unusual for one to encounter strictly identical stimuli, and it becomes increasingly difficult to define what a scan path recapitulation looks like as the differences in viewing conditions and accidental properties increase between the encoding and recognition of a given exemplar.

The present study did not directly test whether the perceptual mechanisms at play during recognition of face images identical to those seen at encoding differ from the mechanisms at play during recognition under more ecological conditions (i.e., of non-identical images). However, we regard it likely that specialized facial recognition mechanisms contribute a greater degree to the successful recognition of even identical facial images than do mere pictorial or other general visual recognition mechanisms. Specifically, in one study [34], participants studied images of faces and of butterflies, and were tested for recognition using identical images. Though the variability in the pictorial image properties was greater across the images of the butterflies than across the images of the faces, participants’ recognition accuracy was much greater for faces than for butterflies, suggesting some difference in how these classes of stimuli were processed for recognition.

### 4.2. Future Directions

Future research is necessary to confirm and better clarify the details of our proposed account of face encoding and recognition and to address some of the limitations of our study. For example, how, if at all, do the small but systematic differences in fixation density distribution that we observed between encoding and recognition in the second ordinal fixation relate to the cognitive processes involved? In particular if initial fixation below the eyes is optimal for face recognition [1], then why was there a relative decrease in fixation over the eye-region during recognition? It is important to consider that the point below the eyes is optimal at the group level, but not necessarily for a given individual. Another prior study [30] revealed that individual observers have idiosyncratic optimal fixation locations that correspond to their idiosyncratic preferred fixation locations during face recognition. Though most individuals in the healthy population prefer to gaze at or near the eyes, a non-negligible proportion prefers to gaze at lower facial features [31]. Therefore, one could speculate that the small increase in fixation density over lower facial features observed during recognition reflects this proportion of observers shift of gaze from the eyes at encoding toward their idiosyncratic optimal fixation location at recognition. However, an individual’s idiosyncratic preferred fixation location during face viewing is similar between face study and test and across time [26], and so such individual differences would not seem to account for our result of differential fixation density patterns between encoding and recognition.

Also, why does the fine-grained pattern of differences in fixation density between encoding and recognition in the second ordinal fixation interact with our stimulus presentation time conditions? Though we found no evidence of a main effect of our stimulus presentation time conditions, some previous research suggests subtle effects of time restriction on eye movement patterns to faces. However, due to differences in paradigm and inconsistency of the results of those studies, it is unclear whether such phenomena could relate to our results. One study [12] reports that initial fixations landed slightly but statistically significantly higher on the face for 350 ms than for 1500 ms stimulus presentation times. Another similar study [30] found highly correlated observer idiosyncratic vertical positions of initial fixations between 350 ms and 1500 ms stimulus presentation times; however, the slope and intercept of the regression suggest that those fixations were slightly lower on the face for 350 ms than for 1500 ms stimulus presentation times. Further, in both those studies, recognition was performed with face identities on which participants had been highly trained, and so the paradigm differs from that of the current study.

While two fixations may suffice for optimal face recognition, several more fixations are necessary for optimal face encoding. Future research is required to determine whether a precise number of fixations might suffice for face encoding, whether other conditions, such as the particular sequence of fixations, affect face encoding, whether fixation on a specific location(s) would influence encoding, and which cortical memory recall systems affect recognition. There is already neuropsychological evidence that the neural substrates for new learning of faces are distinct from those required for the representation of already learned faces [68]. Additionally, if encoding proceeds as an integration of visual feature information to form a face identity representation, the neural basis for this process and how that neural representation is activated so rapidly at recognition warrants elucidation. Given previous evidence that object recognition may share at least some of the neural mechanisms of face recognition [69,70,71,72,73], and given that unitization or holistic processing has been reported also for non-face stimuli such as letters, words, objects, and bodies [74,75], this account of gradual feature integration at encoding for rapid holistic recognition may not be specific to faces, but may, rather, be an important general visual process.

## 5. Conclusions 

Our study investigated the influences of experimental phase (encoding/recognition) and stimulus presentation time (short/long) on eye movements to faces. Our results reveal that eye movement dynamics differ between the encoding and recognition, and provide evidence for distinct perceptual processes between encoding and recognition. Recognition is not achieved through replay of the scan paths made during encoding. Rather, taken together, our results instead suggest that feature information is integrated over many fixations during encoding, but that a representation formed through this integration can be rapidly and holistically activated during recognition within a small number of fixations.

## Figures and Tables

**Table 1:** Outline of the experimental paradigm and counterbalancing of stimulus presentation time blocks. The order of the experimental phases (top row) was the same for all participants. All of the “old” faces in the first two blocks of the test phase were the same set of faces contained within the first block of the study phase (indicated by ‘A’ in the second row). Likewise for the “old” faces in the last two blocks of the test phase and the second block of the study phase (indicated by ‘B’ in the second row). ‘A’ and ‘B’ refer to arbitrary stimulus subsets that differed pseudorandomly across participants. The order of the stimulus presentation time conditions of the blocks was psuedorandomized across participants (last four rows).

Experimental Phase:	Study Phase		Test Phase			
Study/“old” Face Stimulus Subset:	A	B	A		B	
Stimulus presentation time block order 1:	Short	Long	Short	Long	Short	Long
Stimulus presentation time block order 2:	Short	Long	Long	Short	Long	Short
Stimulus presentation time block order 3:	Long	Short	Short	Long	Short	Long
Stimulus presentation time block order 4:	Long	Short	Long	Short	Long	Short

## Supplementary Materials

The following are available online at https://www.mdpi.com/2411-5150/3/1/9/s1, Figure S1: Fixation Durations for the First Ordinal Fixation; Figure S2: Study versus test phase vertical-profile density curves and statistical contrasts for the first ordinal fixation, separated by (A) the short presentation time condition, and (B) the short presentation time condition; Figure S3: Study versus test phase fixation density plots, difference plots, and statistical contrasts for the first ordinal fixation, separated by (A) the short presentation time condition, and (B) the long presentation time condition; Figure S4: Short versus long presentation time condition vertical-profile density curves and statistical contrasts for the first ordinal fixation, separated by (A) study phase, and (B) test phase; Figure S5: Short versus long presentation time condition vertical-profile density curves and statistical contrasts for the second ordinal fixation, separated by (A) study phase, and (B) test phase; Figure S6: Study versus test phase fixation density plots, difference plots, and statistical contrasts for the second ordinal fixation, separated by (A) the short presentation time condition, and (B) the long presentation time condition; Figure S7: Opposing conditions vertical-profile density curves and statistical contrasts for the second ordinal fixation, separated by (A) study phase, short presentation time versus test phase, long presentation time, and (B) study phase long presentation time versus test phase short presentation time; Figure S8: Consistency of Fixation Locations vs. Recognition Performance; Figure S9: Opposing conditions vertical-profile density curves and statistical contrasts for the first ordinal fixation, separated by (A) study phase, short presentation time versus test phase, long presentation time, and (B) study phase long presentation time versus test phase short presentation time; Figure S10: Study versus test phase fixation density plots, difference plots, and statistical contrasts for the third ordinal fixation, separated by (A) the short presentation time condition, and (B) the long presentation time condition, Supplementary Discussion.
