# Supplementary material for: Eye Movement Dynamics Differ between Encoding and Recognition of Faces
Source: Vision (Basel). 2019 Feb 12;3(1):9. doi: 10.3390/vision3010009 (PMC6802769; doi:10.3390/vision3010009)
Supplement: Supplementary file 1 [file vision-03-00009-s001.pdf]

Supplementary Result

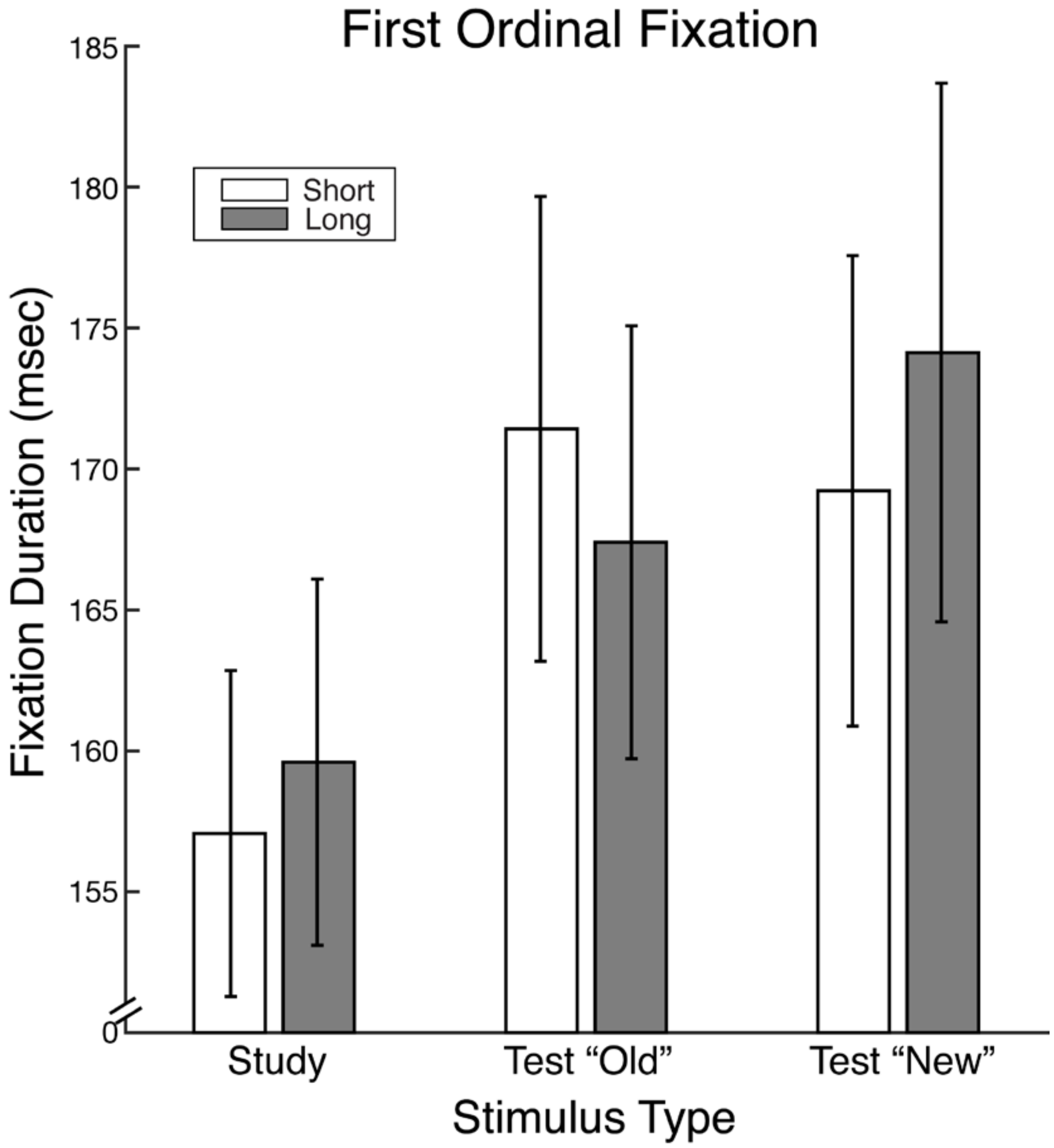

Figure S1. Fixation Durations for the First Ordinal Fixation. The average of the participants' median fixation durations following the first saccade are plotted by stimulus experience type (Study, “old” test face, “new” test face) and stimulus presentation time condition (white bars = “short” one second, gray bars = “long” five seconds).

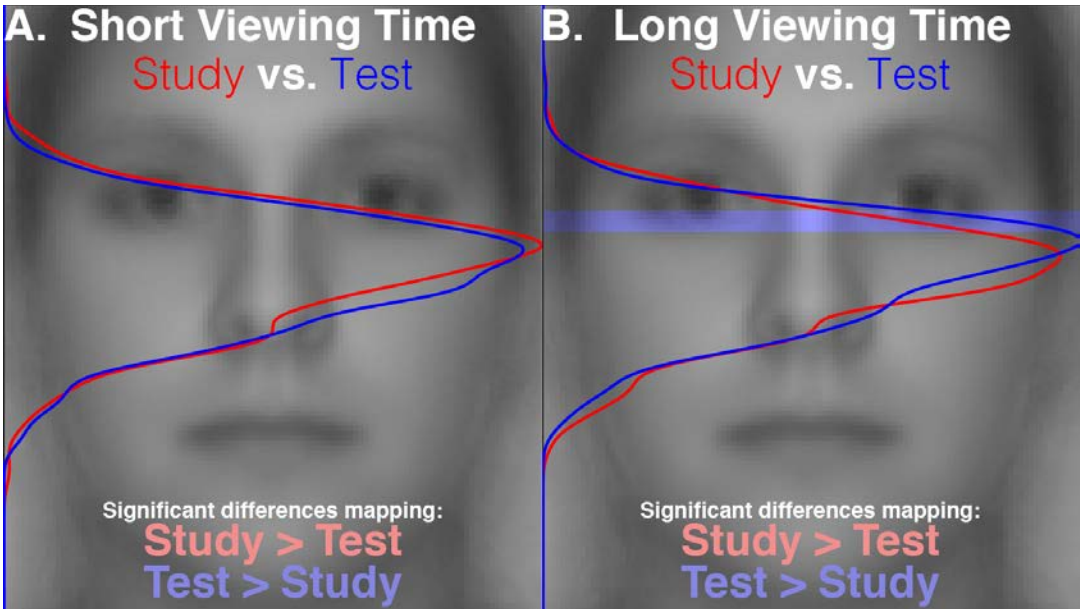

Figure S2. Study versus test phase vertical-profile density curves and statistical contrasts for the first ordinal fixation, separated by (**A**) the short presentation time condition, and (**B**) the long presentation time condition. The face image is highlighted where relative vertical density was significantly different ($q < 0.05$) between the contrasted conditions. Curves are scaled relative to the maximum density between the two given contrasted curves.

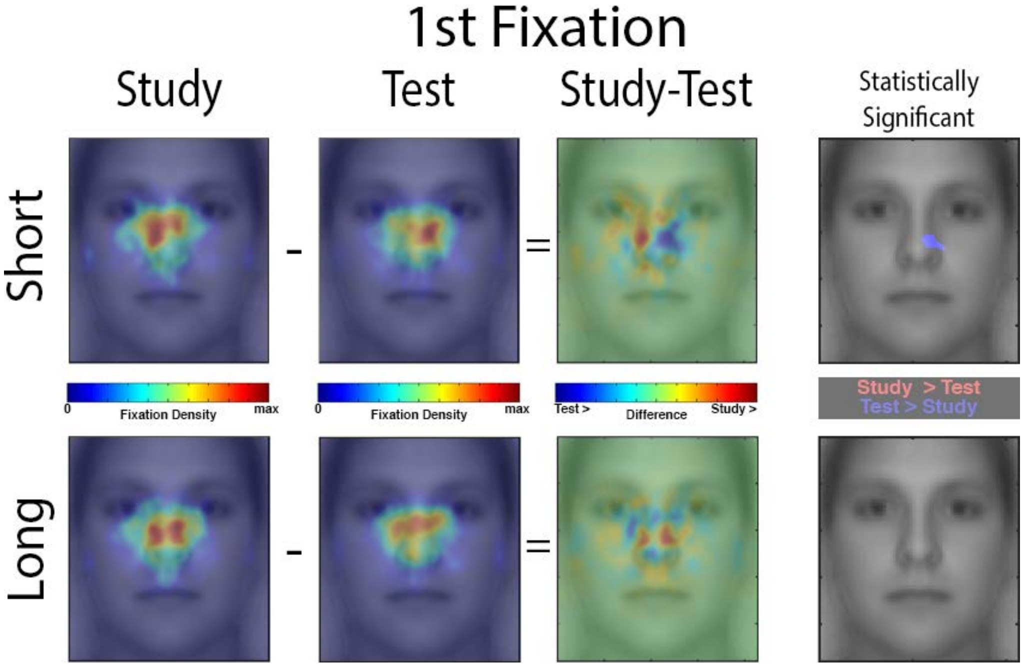

Figure S3. Study versus test phase fixation density plots, difference plots, and statistical contrasts for the first ordinal fixation, separated by (**A**) the short presentation time condition, and (**B**) the long presentation time condition. The face image is highlighted where relative vertical density was significantly different ($q < 0.05$) between the contrasted conditions.

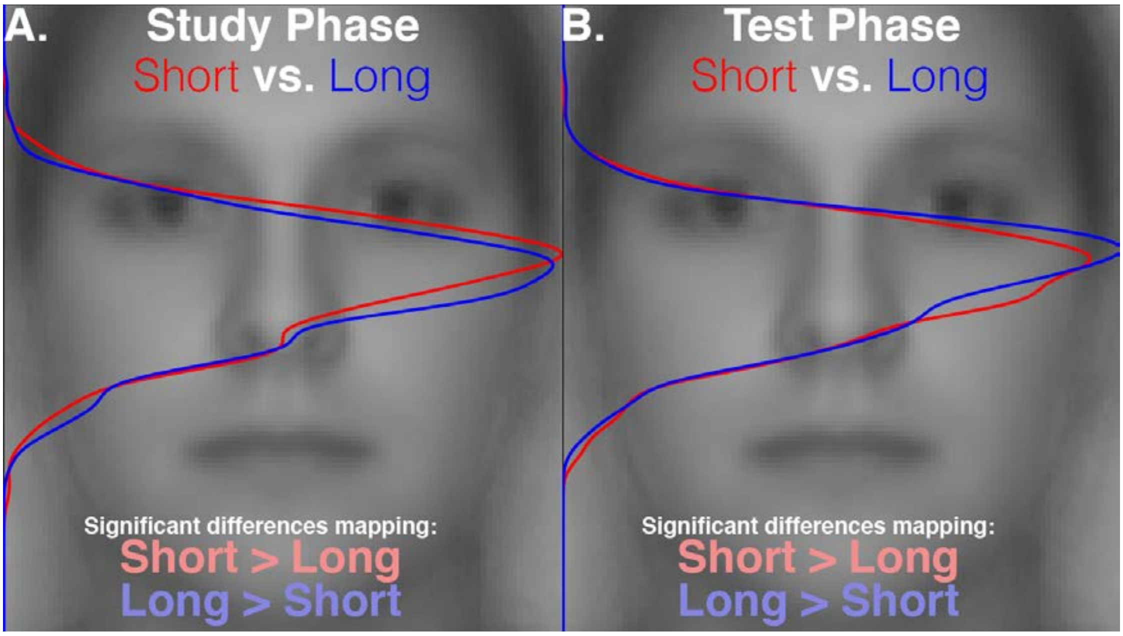

Figure S4. Short versus long presentation time condition vertical-profile density curves and statistical contrasts for the first ordinal fixation, separated by (**A**) study phase, and (**B**) test phase. The face image is highlighted where relative vertical density was significantly different ($q < 0.05$) between the contrasted conditions. Curves are scaled relative to the maximum density between the two given contrasted curves.

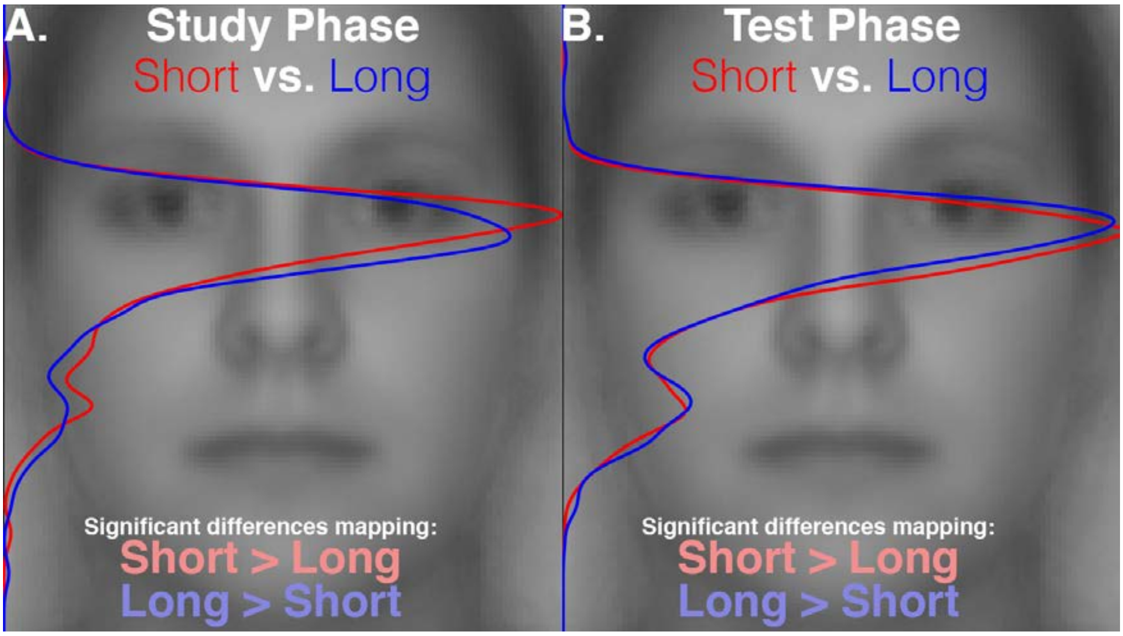

Figure S5. Short versus long presentation time condition vertical-profile density curves and statistical contrasts for the second ordinal fixation, separated by (**A**) study phase, and (**B**) test phase. The face image is highlighted where relative vertical density was significantly different ($q < 0.05$) between the contrasted conditions. Curves are scaled relative to the maximum density between the two given contrasted curves.

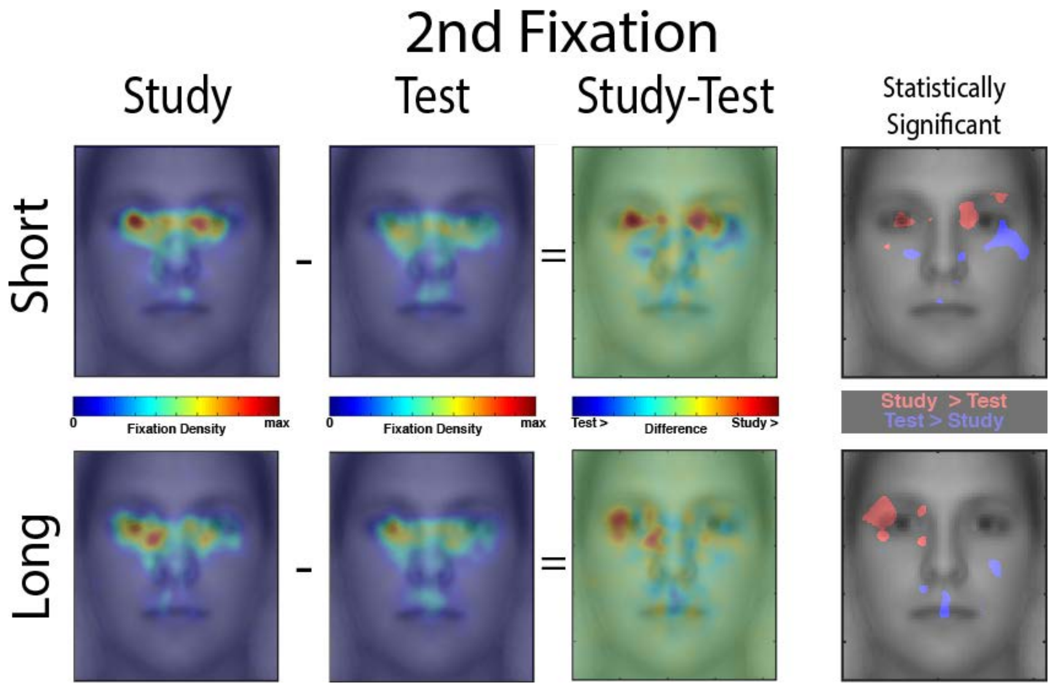

Figure S6. Study versus test phase fixation density plots, difference plots, and statistical contrasts for the second ordinal fixation, separated by (**A**) the short presentation time condition, and (**B**) the long presentation time condition. The face image is highlighted where relative vertical density was significantly different (q < 0.05) between the contrasted conditions.

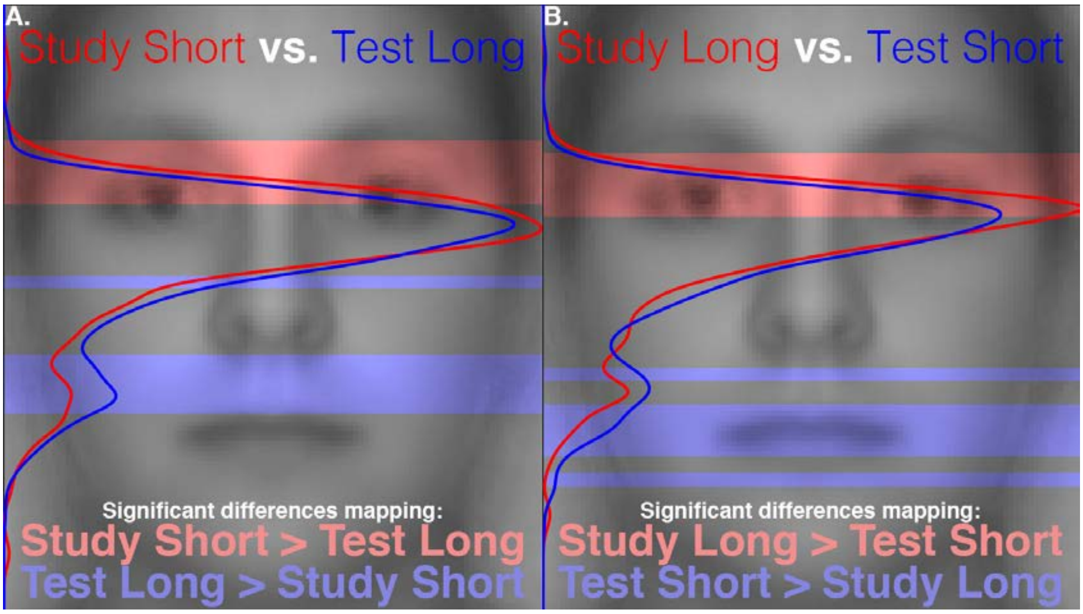

Figure S7. Opposing conditions vertical-profile density curves and statistical contrasts for the second ordinal fixation, separated by (**A**) study phase, short presentation time versus test phase, long presentation time, and (**B**) study phase long presentation time versus test phase short presentation time. The face image is highlighted where relative vertical density was significantly different (q < 0.05) between the contrasted conditions. Curves are scaled relative to the maximum density between the two given contrasted curves.

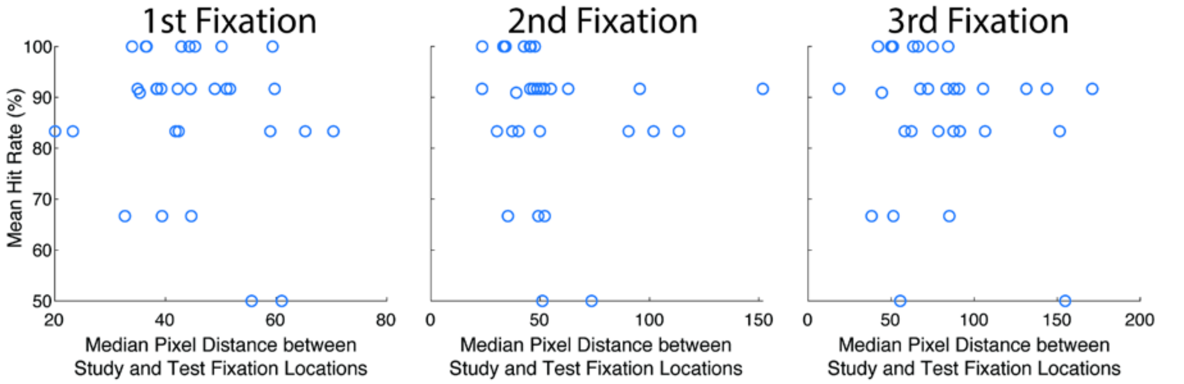

Figure S8. Consistency of Fixation Locations vs. Recognition Performance. This is quantified as each participant's median distance between study and test phase trials' fixation location versus their mean hit rate for those trials. Each of the first three ordinal fixations are plotted separately. The data shown are from the experimental condition where both the study and test phase stimuli had a long presentation time. If the consistency in fixation locations and sequences between study and test phases are functional for facial recognition, then a significant negative correlation between distance and hit rate should be observed. No significant correlations were observed for this condition (all $r(29) < -0.21$, $p > 0.26$) or for the other three conditions (all $r(29) < -0.23$, $p > 0.21$).

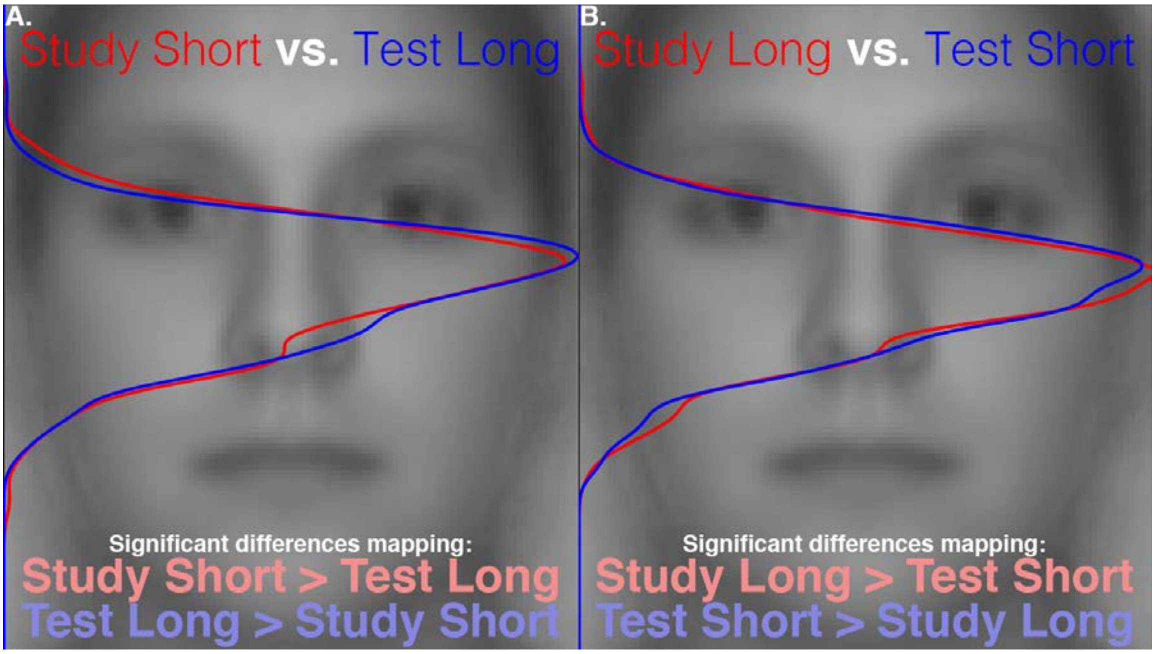

Figure S9. Opposing conditions vertical-profile density curves and statistical contrasts for the first ordinal fixation, separated by (**A**) study phase, short presentation time versus test phase, long presentation time, and (**B**) study phase long presentation time versus test phase short presentation time. The face image is highlighted where relative vertical density was significantly different ($q < 0.05$) between the contrasted conditions. Curves are scaled relative to the maximum density between the two given contrasted curves.

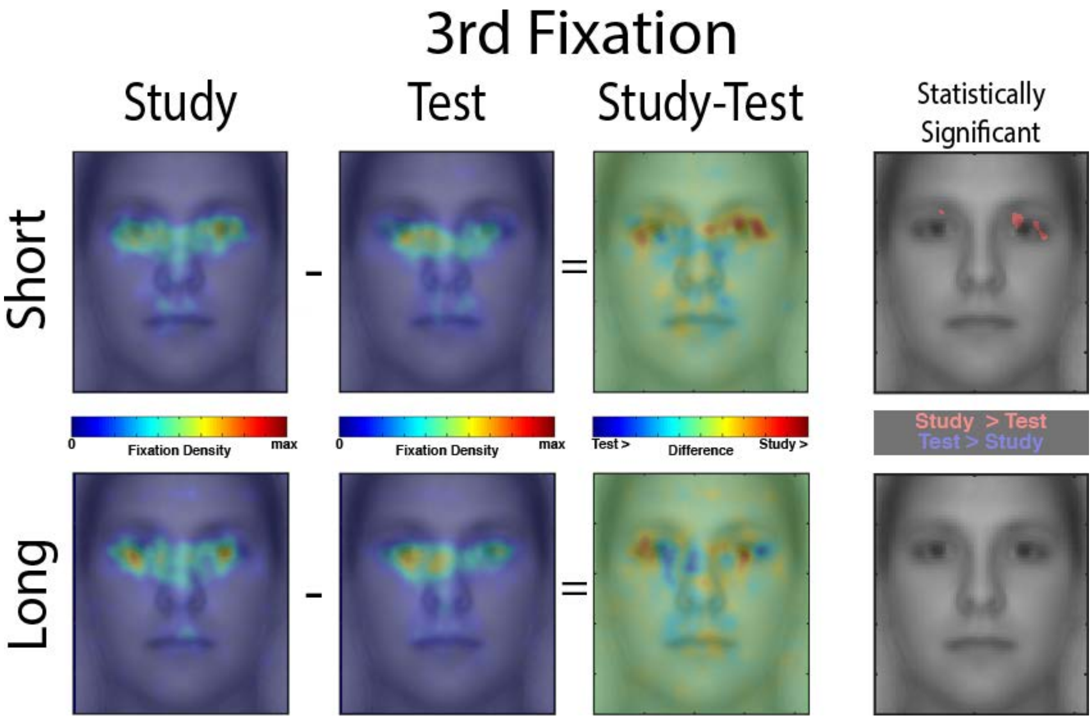

Figure S10. Study versus test phase fixation density plots, difference plots, and statistical contrasts for the third ordinal fixation, separated by (**A**) the short presentation time condition, and (**B**) the long presentation time condition. The face image is highlighted where relative vertical density was significantly different ($q < 0.05$) between the contrasted conditions.

Supplementary Discussion

The main results of two recent individual differences studies have been interpreted in a way that partially contradicts the account of gradual feature integration at encoding for rapid holistic recognition that we propose; however, both the analyses and the interpretation of those two studies can be fundamentally criticized. In these studies by Chuk, Chan, & Hsiao (2017) and Chuk, Crookes, Hayward, Chan, & Hsiao (2017), the dataset utilized was identical and the analyses were similar and related, and so here we discuss both studies together as a single set of results.

Those authors measured eye movements during face learning and recognition in an experimental paradigm highly similar to that of the present study. Then they modeled the spatiotemporal dynamics of each participant's eye movements as Hidden Markov Models (HMMs). Theoretically, in this context, the hidden Markov states represented in the model would correspond to specific regions of the face within which fixations tend to fall and among which fixations tend to transition. The centers and sizes ("emissions") of the hidden Markov states would, respectively, reflect the average location and variance in location of the fixations associated with the given state. Also, prior probabilities for each hidden state would refer to the probability that first fixations to the face begin in each of those facial regions. Transition probabilities would refer to the probabilities that fixation locations transition (or remain) among each pair of those hidden Markov states. The authors chose to produce two or three clusters of participant HMMs in the learning phase and also, separately, in the recognition phase. The resultant clusters were identified as distinct types of eye movement patterns, namely, a "holistic" pattern characterized by hidden Markov states centered over the face center and "analytic" pattern(s) characterized by hidden states centered over the lower part of the eyes, in addition to other hidden states over the face center. Participants whose learning or recognition phase HMM belonged to an "analytic" cluster performed better at recognition, on average, than those belonging to a "holistic" cluster. Further, the similarity of participants' recognition phase HMMs to the representative "analytic" HMM positively correlated with

recognition performance (When three, rather than two, clusters were produced, the left eye-biased "analytic" HMM specifically correlated with performance). Finally, no evidence was found that changing pattern type between learning and recognition was related to recognition performance. Thus, taken together, the results were interpreted as indicating that analytic eye movement patterns at learning and/or recognition of faces leads to better recognition performance.

Fundamental aspects of those studies' analyses can be called into question though. To begin with first principals, the (undemonstrated) assumption underlying modeling spatiotemporal dynamics of eye movements as HMMs is that eye movements are Markov processes, or, in other words, that the length into an eye movement sequence does not influence the probability of one's gaze transitioning from a given region to the next. However, evidence from those authors' own analyses clearly reveal that eye movements are not Markov processes. Specifically, in one analysis on the recognition phase data, HMMs were produced separately for fixations 1-3, fixations 4-6, and all remaining fixations. Each HMM was classified as one belonging to one of the three types ("holistic", left eye biased "analytic", and right eye biased "analytic") that had previously been produced as HMMs derived from all the fixations together. They found that the proportions of participants' HMMs belonging to each cluster type significantly ($p = 0.01$) differed in the first three fixations compared to those in all fixations together. Further, most (26/48) participants' data for the first three fixations yielded HMMs classified as a left eye biased "analytic"; however, about half of those classifications changed to "holistic" for the later fixations. If eye-movements are Markov processes as the authors assumed, then the HMMs for different numbers of fixations into fixation sequences should have been identical with respect to hidden Markov state locations and sizes and of the transition probabilities among the states (the prior probabilities could differ). On the contrary, the results prove that their assumption is false.

If eye movements are not Markov processes, then differences among HMMs of eye movement data are not easily interpreted. For example, those authors report that even the representative "analytic" HMM from their learning phase data was statistically significantly different from the representative "analytic" HMM from the recognition phase, and likewise for between representative "holistic" HMMs, revealing that models they identified as the same on qualitative grounds were, statistically speaking, actually not. In addition, their learning phase data included about 14 fixations on average (or 5 seconds exactly), whereas their recognition phase data included only about 6 fixations on average (or 1.84 seconds on average). Thus, analytic artefacts analogous to those illustrated in Figure 1 of the main manuscript are also possible in the comparison of HMMs modeled using non-Markovian eye movements of different sequence lengths. These factors likely explain why, in a joint analysis of the learning and recognition phases, such a large proportion (40%) of their participants' HMMs changed cluster membership ("analytic" or "holistic") from the learning to the recognition phase, while such changes also had no apparent relation to recognition performance.

Relatedly, if only the first two fixations are necessary for optimal recognition (Hsiao & Cottrell, 2008), then for most of their critical analyses they included mostly functionally superfluous fixations in the data for the recognition phase, thus at least partially obscuring functionally relevant eye movement dynamics in any case. Indeed, when recognition phase HMMs were produced separately for fixations 1-3, fixations 4-6, and the remaining fixations, only for fixations 1-3 did the participants whose models were classified as left eye biased "analytic" significantly outperform those whose were classified as another model type.

Furthermore, the number of clusters produced for each analysis was not discovered in a data-driven way as natural clusters. Rather, the number of clusters was imposed a priori, and so the "analytic" and "holistic" HMM clusters are artificial partitions of their data. While these clusters were reported as statistically significantly different from one another, this should not be taken as indication that the clusters were truly distinct from one another. To clarify this with an analogy, one could take a normally distributed sample of data and conduct an independent samples t-test between the data above the mean and the data below the mean. This would yield a statistically significant difference, even though no natural separation exists between these two groups of the data (i.e., no bimodality in

the full sample). Likewise, the statistically significant differences in "distance" from the different types of representative HMMs follow merely from the fact that the HMMs were partitioned with the same criteria by which they are compared. True natural clustering would require that some separation of the clusters be evident and shown to be quantitatively optimal compared to other possible cluster number solutions (including the one cluster solution).

Nonetheless, even if one ignores all of these analytic issues and takes at face value the representative HMMs of the clusters that resulted from their analyses, identifying one/some cluster(s) as "analytic" and the other as "holistic" is very much disputable. Rather, the labels could, arguably, be reversed. Those authors' basis for labeling their "holistic" cluster as such was that all the hidden Markov states of the representative HMM were centered over the face center. However, as was apparent in accompanying heatmaps, the centers of the hidden states did not represent typical fixation locations. The emissions of the hidden states were spatially expansive, covering most, or all, of the face, reflecting that typical eye movements were widely dispersed over the face, and were, overall, more dispersed than those of the "analytic" cluster(s). So, then their interpretation that the "holistic "cluster is characterized by fixations to the center of the face is incorrect, and the widely-dispersed pattern of eye movements could reasonably, rather, be considered an analytic pattern, since more features are sampled by this group. Further their representative "analytic" cluster(s) were characterized by the presence of hidden states centered, with spatially-restricted emissions, over the lower parts of each eye. From this, and accompanying heatmaps, it can be inferred that typical eye movements for those clusters were, indeed, restricted to the regions just below the eyes. However, as mentioned in the introduction, that fixation location has been shown to be functionally optimal for holistic processing of faces (Peterson & Eckstein, 2012), and so their "analytic" cluster(s) could, instead, reasonably be interpreted as representing a holistic pattern. Given prior evidence that individual differences in holistic processing are related to face recognition ability (DeGutis, Cohan, Mercado, Wilmer, & Nakayama, 2012; DeGutis, Mercado, Wilmer, & Rosenblatt, 2013; DeGutis, Wilmer, Mercado, & Cohan, 2013), it should be expected, then, that eye movement patterns more similar to those authors' so-called "analytic" representative HMMs are associated with better face recognition. This is because that pattern of eye movements could be expected to enable more effective holistic processing of faces.
